# Acute Central Serous Chorioretinopathy Outbreak during the COVID-19 Pandemic: A Pilot Study

**DOI:** 10.3390/medicina60010122

**Published:** 2024-01-09

**Authors:** Tal Yahalomi, Yael Sara Pikkel, Roee Arnon, Michael Kinori, Keren Wood, Joseph Pikkel

**Affiliations:** 1 Department of Ophthalmology, Samson Assuta Ashdod Hospital, Faculty of Health Sciences, Ben-Gurion University of the Negev, Be’er Sheva 8410501, Israel; roee.arnon@gmail.com (R.A.); michaelkinori@gmail.com (M.K.); kewoood@gmail.com (K.W.); yossefp@assuta.co.il (J.P.); 2 Rambam Health Care Campus and Faculty of Medicine, Technion—Israel Institute of Technology, Haifa 3109601, Israel; yaelpikkeligal@gmail.com

**Keywords:** central serous chorioretinopathy, CSCR, COVID-19, coronavirus

## Abstract

*Background and Objectives*: This study aims to investigate the potential association between the COVID-19 pandemic and a new presentation of central serous chorioretinopathy (CSCR). *Materials and Methods*: A retrospective analysis was conducted, comparing the incidence of new-onset CSCR cases among ophthalmology patients in a regional medical facility in southern Israel between two distinct periods: the COVID-19 pandemic era in Israel, which occurred from 27 February 2020 to 20 December 2020, and the non-pandemic period from calendar years 2018 to 2021, excluding the specific epidemic phase mentioned. Disease severity was evaluated based on recovery time, visual acuity loss, and central macular thickness via OCT. *Results*: Over the four-year period, 35 new cases of CSCR were recorded. During the COVID-19 pandemic, 17 new cases (0.005% per population) were identified, compared with 18 new cases (0.002% per population) in the preceding three years. The odds ratio for acute CSCR during the pandemic was 2.83 (95% CI, 1.46–5.50) with a *p*-value of 0.02. CSCR cases during the pandemic seemed to exhibit worse clinical characteristics, though not statistically significant. Additionally, 22.2% of the COVID-19 pandemic group had confirmed COVID-19 cases, which was statistically significantly higher than the general population’s reported cases (6%). *Conclusion*: The study revealed a statistically significant increase of over 2.5 times in acute CSCR incidence during the COVID-19 pandemic compared with non-pandemic periods. The findings suggest that the pandemic’s stressful changes may have unintended consequences on the occurrence of CSCR, highlighting the importance of mental health support and psychoeducation for affected patients.

## 1. Introduction

Central serous chorioretinopathy (CSCR) is a relatively common idiopathic disease characterized by unilateral loss of vision, particularly in young healthy men aged 20–50 [1]. This disease is characterized by serous separation of the neurosensory retina in the macular region, which in some cases is accompanied by separation of the retinal pigment epithelium (RPE) layer and may progress to localized atrophy. CSCR is a relatively prevalent disease in North America, with a case incidence of 9.9 per 100,000 men and 1.7 per 100,000 women [2]. Although the pathophysiology of the mechanism causing this separation is not well established, multiple risk factors have been identified, with the most important being an increase in steroid levels, both endogenous and exogenous. Another feature is the relation to Type A personality, which is characterized by accomplished people such as engineers, physicians, pilots, and others whose blood glucocorticoid levels are high due to the stress associated with this personality type. CSCR typically resolves on its own within two to three months, and follow-up is usually the standard of care [3]. However, treatment is generally recommended for persistent or recurrent cases or for patients with occupational demands [4]. The choroidal hyperpermeability observed in CSCR could be targeted via a photodynamic therapy (PDT) that uses verteporfin, a photosensitizer that builds up in vessels causing endothelial damage and vascular hypoperfusion. Previous reports have demonstrated that PDT can be utilized to lower SRF and improve visual outcomes in patients with chronic CSCR [5]. Reduced-fluence PDT was found to be more effective in improving visual outcomes and reducing central retinal thickness than intravitreal ranibizumab, according to an RCT study by Bae et al. [6]. In comparison with half-fluence PDT, half-dose PDT on eyes with chronic CSCR indicated that the former allowed for faster SRF resolution and decreased recurrence [7]. Other treatment options include the use of a mineralocorticoid receptor antagonist, such as eplerenone, for five weeks, as originally recommended by Zhao and Bousquet [8]. They have reported a significant prompt improvement in retinal detachment along with improvements in mean central macular thickness, sub-retinal fluid height, and visual outcome. These improvements continued at the five-month follow-up [9]. Other varieties of treatments have been examined including spironolactone, finasteride, melatonin, and acetazolamide, and although some effectiveness has been shown, these treatments have been limited by many side effects and only partial success [8,10,11,12]. A case study of a patient treated with rifampin for two years following developing multifocal CSCR and persistent SRF revealed total remission of SRF in merely one month [13]. Hepatotoxicity, anorexia, and orange-colored bodily secretions are some of the side effects of rifampin [13]. Melatonin is an endogenous neuromodulator that has been connected through an antioxidant mechanism to circadian cycles, sleep regulation, aging, and neuroprotection [14]. Melatonin was considered an attractive option for CSCR treatment since it has a low side effect profile and has the ability to inhibit the action of corticosteroids. Regarding visual acuity and CMT, a study that included eight patients demonstrated noteworthy gains in comparison with controls [15]. Moreover, there were no adverse effects noted. Anti-vascular endothelial growth factor (VEGF) medications are used on the assumption that choroidal disease may raise VEGF levels. Nevertheless, research has revealed that VEGF levels in CSCR patients and control individuals are comparable [16]. At six months, there was no difference in visual acuity or CMT between patients treated with intravitreal bevacizumab and those in a control group, according to a meta-analysis of several randomized controlled trials [17]. It is notable that anti-VEGF injections are beneficial for patients in which CSCR and concurrent choroidal neovascularization are present [18]. Metoprolol tartrate, a selective beta-1 blocker, and metipranolol, a non-selective beta blocker, have both been demonstrated in several other studies to result in CSCR resolution [19]. By comparison, there was no difference in the duration of acute CSCR between the beta-blocker group and the control group in a randomized controlled trial [20]. The study examined individuals with acute CSCR; therefore, patients with chronic CSCR may not find the conclusions to be as easily relevant. Despite some studies’ evidence of the benefit of anti-adrenergic medications, they have not been routinely used in the treatment of CSCR.

Since CSCR is mainly caused by high levels of glucocorticoids, Type A personality, and psychological stress, previous studies have suggested that patients with CSCR could benefit from psychoeducation, support, or psychological intervention that has been shown to alleviate anxiety and stress [21,22].

During the COVID-19 pandemic in 2020, we have observed an assumed rise in the number of patients presenting with acute CSCR to the local medical center, either being admitted to the emergency room or being referred to the clinic for retinal specialists. This presumption led us to plan an investigation into the growth in acute CSCR incidence. In this study, we wish to examine the association between the COVID-19 pandemic and the incidence and severity of CSCR compared with non-pandemic times. The study aimed to provide valuable insights into the characteristics and outcomes of CSCR cases during the specified periods and to identify potential associations or differences related to the pandemic era.

## 2. Materials and Methods

### 2.1. Study Design

This retrospective observational cross-sectional study was conducted at Samson Assuta Ashdod Hospital, the regional hospital in southwestern Israel. Public health care is the standard practice in Israel, notably in the southwest region. Once CSCR is suspected, patients are mostly referred to a retinal specialist located in a public hospital. The sole regional hospital in southwestern Israel that is the subject of this study serves a population of around 300,000 people. This study was approved by the Institutional Review Board (IRB) of the hospital, and all data collection and processing procedures were performed in accordance with the guidelines and norms outlined in the Declaration of Helsinki, which governs ethical principles in medical research involving human subjects.

### 2.2. Participants

This study included patients aged 18 to 90 years who were diagnosed with a new presentation of central serous chorioretinopathy (CSCR) and were admitted to Samson Assuta Hospital between the years 2018 and 2021. Patients were identified either through their presentation in the emergency room or referral to the hospital’s retinal specialistclinic.

### 2.3. Exclusion Criteria

Patients with concurrent retinal or ocular conditions that could potentially influence visual acuity, a history of corticosteroid use, or previous episodes of CSCR were excluded from the study. Patients who did not experience a disease remission for more than a year were classified as chronic CSCR patients and were also excluded from the study.

### 2.4. Data Collection

Data for this study were collected retrospectively from the medical records of eligible patients. Demographic information, systemic and ocular history, visual acuity at presentation, and central macular thickness (CMT) were recorded as part of the data collection process. Visual acuity was assessed using the standardized Snellen acuity chart and converted to LogMAR units for analysis. Central macular thickness measurements were obtained using SD-OCT (OCT-HS100, Canon Inc., Tokyo, Japan). COVID-19 diagnosis confirmation was assessed based on the medical records and confirmed via PCR (Polymerase Chain Reaction) blood test.

### 2.5. Diagnosis Confirmation

A single experienced retinal specialist (J.P.) reviewed and confirmed the diagnosis of CSCR for each patient included in the study. The diagnosis was established based on a clinical examination and using macular SD-OCT (OCT-HS100, Canon Inc., Tokyo, Japan). A further ultra-widefield fluorescein angiography (Canon Inc., Tokyo, Japan) was carried out when there was concern regarding the differential diagnosis.

### 2.6. Protocol for Treatment and Follow-Up

Observation was the standard of care for newly presented patients because CSCR typically resolves spontaneously within two to three months [3]. Patients were monitored one and three months following their initial presentation, and if they continued to exhibit symptoms three months later, they were treated with a mineralocorticoid receptor antagonist, such as eplerenone, for five weeks, as originally recommended by Zhao and Bousquet [8].

### 2.7. Comparison of Periods

The study aimed to compare CSCR cases between two distinct periods: the pandemic era in Israel, which was defined from 27 February 2020 to 20 December 2020, and the non-pandemic era including the two periods from 1 January 2018 to 26 February 2020 and 21 December 2020 to 31 December 2021. With regard to the study’s geographic location, the pandemic era in Israel was defined from 27 February 2020, which marked the first disease outbreaks, the imposition of travel restrictions between and within towns, the shutdown of most businesses, the closure of schools and other academic educational institutions, and protracted periods of nationwide lockdowns. Israel went through a severe economic and social crisis, and many people lost their jobs or enterprises. That period ended rapidly with the establishment of the national vaccination program, which began on 20 December 2020 and marked the beginning of Israel’s social and economic recovery. Israel has administered the highest doses of vaccines per population worldwide. This initial phase of vaccination had clearly been rapid and effective, earning Israel its fast restoration. These regional geopolitical differences were taken into consideration while defining the pandemic era for this study [23]. The objective was to evaluate potential differences in CSCR cases and outcomes during these different timeframes.

### 2.8. Statistical Analysis

Categorical variables were summarized as frequencies and percentages. Continuous variables were evaluated for normal distribution using a histogram. Although none were found to have a normal distribution, continuous variables were reported as mean and standard deviation for the convenience of the reader. To compare variables between different time periods, the Fisher exact test was used for categorical variables and the Mann–Whitney U test was used for continuous variables. The Chi-square test was used to compare rates between the two time periods. The incidence rates were determined by dividing the number of cases by the estimated 300,000 people living in the southwestern region of Israel. All statistical tests were two-sided and *p* < 0.05 was considered statistically significant. NCSS was used for all statistical analysis (NCSS 2022 Statistical Software version 22.0 (2022), NCSS, LLC., Kaysville, UT, USA).

### 2.9. Sample Size

The sample size was calculated taking into account a ratio of 1:3 COVID-19: non-COVID-19 patients. We estimated that the ratio would be 5.8 per 100,000 population in the non-COVID-19 era while it would be twice the incidence in the COVID-19 epidemic era. Therefore, approximately 315,000 samples were needed in the COVID-19 era.

## 3. Results

On average, 2500 patients are admitted to the local hospital’s ophthalmology emergency department annually. In the COVID-19 era, the number of patients admitted was approximately 1450 patients. Two patients with type 2 diabetes did not exhibit any clinical signs of diabetic retinopathy or a characteristic imaging pattern of diabetic macular edema via macular OCT. One patient had well-managed systemic lupus erythematosus without a history of ocular involvement or current ocular inflammatory disease. Clinical and several imaging modalities ruled out differential diagnosis.

Over the four-year study period, 35 new cases of CSCR were presented to the emergency department or were referred to the regional retinal specialist clinic. Of these, 29 (82.8%) were men and 6 (17.1%) were women. The mean age of the patients was 44.7 ± 10.1 years, six (17.1%) had myopia, four (11.4%) had a history of refractive surgery, and one was pseudophakic. Contralateral eye disease was present in two patients (5.7%) from the COVID-19 group.

The two groups were similar in demographic, systemic, and ocular background (Table 1).

**Figure 1:**
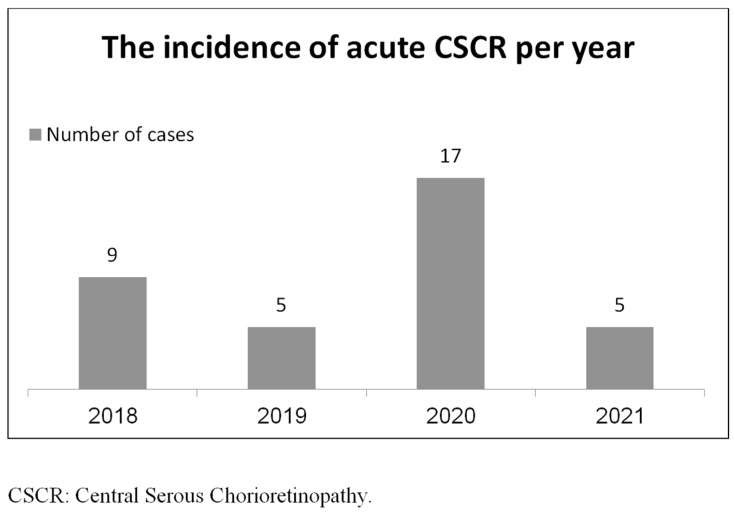
The incidence of acute CSCR per year.

The retrospective data chart of all study participants is presented in the Supplementary Materials, Table S1. During the COVID-19 pandemic era, there were 17 new cases of CSCR (0.005% per population). In comparison, there were 18 new cases of CSCR over the comparative three-year period (0.002% per population) (Figure 1).

**Figure 2:**
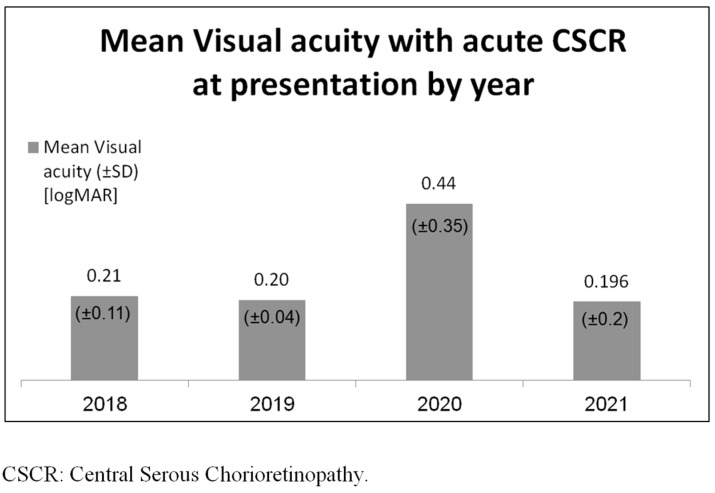
Mean visual acuity with acute CSCR at presentation.

The odds ratio for an acute attack of CSCR during the COVID-19 pandemic compared with the non-pandemic period was found to be 2.83 (95% CI, 1.46–5.50), with a probability value of 0.02. When comparing the acute attack of the CSCR COVID-19 outbreak group to the non-COVID-19 group, the visual acuity upon presentation was 0.4 and 0.2 LogMar, respectively (*p* = 0.1). Further results are presented in Figure 2.

**Figure 3:**
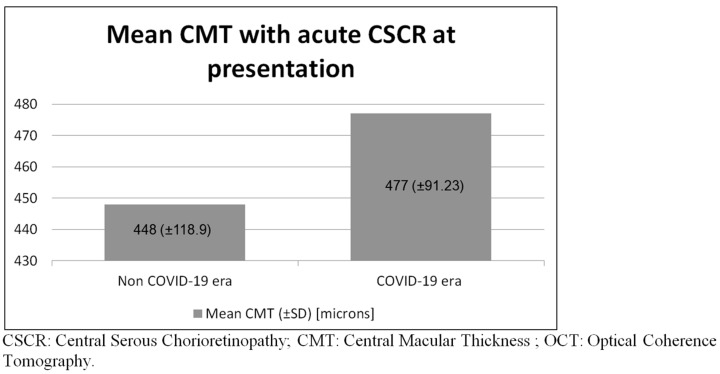
Mean central macular thickness with acute CSCR at presentation observed via Macular OCT.

For the COVID-19 epidemic outbreak group compared with the non-COVID-19 group, the initial presentation and hospital administration times were delayed by 33.5 and 22.1 days, respectively (*p* = 0.84). As shown in Figure 3, the initial CMT at presentation for the COVID-19 outbreak group compared with the non-COVID-19 group was 477.7 and 501.6 microns, respectively (*p* = 0.68).

The reported time to remission for the disease was 6.2 months for the COVID-19 pandemic group and 4.1 months for the non-COVID-19 group (*p* = 0.08) (Table 2).

There were five cases (22.2%) of confirmed COVID-19 reported for the COVID-19 epidemic outbreak group. We found a statistically significant higher prevalence of newly diagnosed patients with a confirmed PCR test of COVID-19 in the CSCR COVID-19 outbreak group compared with the reported cases in the general population during the same period, as reported by the Israel Science and Technology Directory [24] (22.2% vs. 6%, respectively, (*p* = 0.0135)).

## 4. Discussion

This is the first study to discover a potential positive relationship between the incidence of CSCR and the COVID-19 pandemic eras.

On 11 March 2020, the World Health Organization (WHO) declared the coronavirus disease (COVID-19) to be a pandemic [25]. As of 26 June 2022, there have been over 77 million reported cases globally, resulting in more than 1,000,000 deaths [26]. Coagulopathy and disseminated intravascular coagulation (DIC) have been recognized as frequent causes of death in severe cases of COVID-19 [27,28,29].

As of now, reports of ocular manifestations have included conjunctivitis [30], retinal microvascular changes like retinal microangiopathy [31], cotton wool spots and microhemorrhages [32], acute middle maculopathy and acute macular neuroretinopathy [33], papillophlebitis [34], and central retinal vein occlusion [35].

Multiple risk factors for CSCR have been identified, with the most important being an increase in steroid levels, both endogenous and exogenous. Previous research has suggested that individuals with CSCR may benefit from psychoeducation, support, or psychological intervention to reduce anxiety and stress, as CSCR is primarily associated with high levels of glucocorticoids, Type A personality traits, and psychological stress [21,22].

The COVID-19 pandemic has caused economic and social devastation as a result of attempts to eradicate and quarantine the disease. The pandemic triggered the world’s worst economic downturn in history, with over a third of the world’s population being quarantined at the time [36]. On April 7th, the International Labor Organization expected a 6.7 percent drop in working hours worldwide during the second quarter of 2020, equating to 195 million full-time workers. They also reported that 30 million jobs were lost in the first quarter of 2020, surpassing the 25 million lost during the financial crisis of 2008 [37]. Recent studies have shown an increase in depression, anxiety, insomnia, and suicide rates as a consequence of the COVID-19 pandemic [38,39,40,41]. Psychological stress has been recognized as one of many risk factors that might lead to CSCR, and it has been suggested that this aspect is crucial [21,22]. The association between the COVID-19 pandemic era and the rise of acute CSCR incidence could be explained by the unintended consequences of the incredibly stressful changes in the global economy, society, health, death rate, and unemployment. The importance of comprehending the influence of a patient’s mental health in ophthalmology may be highlighted by this study, as well as the importance of referring patients to receive psychoeducational treatment. In a prior study, the clinical impact of COVID-19 on chronic CSCR patients was evaluated using a retrospective chart review. The clinical measures were compared with those of chronic patients prior to the onset of COVID-19, and no appreciable changes were found in their clinical status, vision, central subfield thickness, subretinal fluid, or pigment epithelial detachment [42]. However, this study focused on the incidence and clinical severity of acute CSCR episodes and discovered that there was a statistically significant rise in the incidence of these cases during the COVID-19 pandemic compared with previous times, while disease severity also worsened, although not statistically significant. All patients who were admitted to the ophthalmologic emergency department or who were referred to the retinal specialist’s clinic were included in this study. Since the medical center under examination is the only regional medical center with a retinal specialist clinic, it is reasonable to presume that a substantial population of patients with acute CSCR in the region was included in this study. During the COVID-19 era, the underlined systemic or ocular illness conditions may have raised the physical or mental burdens on patients. These underlying conditions may have contributed significantly to patients experiencing a more stressful quality of life during the epidemic or act as confounding factors. To provide a more comprehensive look at the effects of the COVID-19 era on patients, it was necessary to consider and incorporate these relevant factors in the analysis. In this study, the demographic, systemic, and ocular baseline characteristics between the two groups were found to be similar. Moreover, we found a statistically significant higher prevalence of newly diagnosed patients with a confirmed PCR test of COVID-19 in the CSCR COVID-19 outbreak group compared with the reported cases in the Israeli general population updated for those times examined. Additionally, there was a tendency toward longer and more severe disease in the COVID-19 pandemic era but it did not reach statistical significance. It is possible that with larger groups of patients, significance would have been reached. The longer duration between initial presentation and admission to the emergency department or referral to the retinal specialist clinic in the COVID-19 group vs. the non-pandemic period (33.5 vs. 22.1 days) could be attributed to people’s fear of being admitted to the hospital during a pandemic, which could be another explanation for the severity of the disease in the COVID-19 group. Cortisol is considered a key element in any acute stress including a severe viral infection. It was discovered that cortisol levels were much higher in COVID-19 individuals and even linked with the infection’s severity [43,44]. Previous studies have indicated that CSCR patients had greater cortisol levels in a similar pattern [45]. This may also be a contributing factor to the rising incidence of CSCR observed in the general population during the COVID-19 pandemic. It is important to note that this study did not directly examine the association between individuals with CSCR and cortisol levels of patients. This limitation should be considered when interpreting the findings of this study. Several other limitations should also be acknowledged. Firstly, this study included a relatively small sample size, which holds limited statistical significance despite covering a four-year period and encompassing data from a region with an approximate population of 300,000 individuals in southwestern Israel. It is essential to carry out additional investigations with a larger sample size and perhaps a multicenter study. Secondly, this study excluded patients who were lost to follow-up and only used data collected from the hospital. Additionally, the follow-up period might not have been long enough to assess for chronic CSCR. This study aimed to compare the disease’s incidence rates during pandemic and non-pandemic periods. Since the goal of this study was not to demonstrate the disease’s longitudinal trend, a time series analysis was not performed. The annual incidence was the same in the years before the epidemic. Lastly, the retrospective design of this study introduces potential biases that should be taken into consideration when interpreting the results. It is necessary to discuss some possible cofounders. In contrast to other periods, our analysis indicates that during the COVID-19 pandemic, fewer individuals were admitted to the ophthalmology emergency department. This can be attributed to the widespread dread of infection. Nonetheless, we cannot completely rule out the possibility that patients became more self-conscious at this time and thus avoided medical examination. The majority of COVID-19 patients in Israel, particularly those in the youthful, middle-aged demographic that comprised this study sample, had a self-limiting illness that allowed them to remain in their homes under quarantine without the need for medical attention or special treatment. Any patient admitted to the hospital was inquired about any past usage of corticosteroids. Patients who had taken exogenous corticosteroids were excluded in order to study the association between the COVID-19 disease and the suspected cortisol or stress levels, which could affect the development of CSCR. There have been reports of a number of ocular adverse effects following the administration of the COVID-19 vaccine. These include subretinal fluid [46], acute macular neuroretinopathy [46], paracentral acute middle maculopathy [46], and Vogt–Koyanagi–Harada syndrome [47]. It is important to note that the establishment of the national immunization program in Israel, which started on December 20, 2020, marked the end of the COVID-19 era that was evaluated in this study. Hence, this did not affect the prevalence of retinal abnormalities to the extent that it constituted a cofounder impact on the COVID-19 group examined in this study.

In conclusion, our study observed a significant increase of over 2.5 times in the incidence of acute CSCR during the COVID-19 pandemic compared with non-pandemic times. This effect could possibly be explained by the unintended consequences of the incredibly stressful changes in the global economy, society, health, death rate, and unemployment, and perhaps emphasizes the importance of mental health, psychoeducation, and support in patients with CSCR.

## Figures and Tables

**Table 1:** Baseline characteristics of patients with acute CSCR.

	COVID-19 Pandemic Era (2020)	Non-COVID-19 Pandemic Era (2018, 2019, 2021)	*p* Value
CSCR incidents per population (per 1000 persons)	17 (0.005)	18 (0.002)	0.02
Female-Number (%)	2 (11.7%)	4 (22.2%)	0.6
Male-Number (%)	15 (88.2%)	14 (77.7%)	
Age-Mean (SD, range)	42.8 (±3.6, 26–64)	46.6 (±9.8, 33–70)	0.2
Ocular medical history			
Myopia (%)	5 (29.4%)	1 (5.5%)	0.08
Previous refractive surgery (%)	3 (17%)	1 (5.5%)	0.33
Pseudophakia (%)	0 (0.0%)	1 (5.5%)	>0.99
Glaucoma (%)	0 (0.0%)	1 (5.5%)	>0.99
Systemic medical history			
Ischemic heart disease (%)	1 (5.8%)	2 (11.1%)	>0.99
Hypertension (%)	0 (0.0%)	2 (11.1%)	0.22
Hyperlipidemia (%)	0 (0.0%)	1 (5.5%)	0.48
SLE (%)	0 (0.0%)	1 (5.5%)	>0.99
BPH (%)	1 (5.8%)	0 (0.0%)	0.48
Hyperlipidemia (%)	0 (0.0%)	1 (5.5%)	0.48
ADHD (%)	0 (0.0%)	1 (5.5%)	0.48
Previous malignancy (%)	1 (5.8%)	1 (5.5%)	>0.99
DM2 (%)	1 (5.8%)	1 (5.5%)	>0.99
Active Smoking (%)	4 (23.5%)	1 (5.5%)	0.11

CSCR: Central serous chorioretinopathy; SLE: systemic lupus erythematosus; BPH: benign prostatic hyperplasia; ADHD: attention-deficit hyperactivity disorder; and DM2: diabetes mellitus type 2.

**Table 2:** Clinical parameters of patients with acute CSCR.

	COVID-19 Pandemic Era (*n* = 17)	Non-COVID-19 Pandemic Era (*n* = 18)	*p* Value
Mean visual acuity at presentation (SD) (LogMar)	0.43 (±0.36)	0.20 (±0.13)	*p* = 0.1
Mean time lag between initial presentation and hospital administration (SD) (days)	33.58 (±10.49)	22.16 (±20.69)	*p* = 0.84
Mean CMT at presentation (SD) (microns)	477.70 (±96.17)	501.62 (±134.05)	*p* = 0.68
Mean Disease remission (SD) (months)	6.2 (±0.60)	4.1 (±0.72)	*p* = 0.08

*p*: Probability value; CSCR: central serous chorioretinopathy; and CMT: central macular thickness.

## Data Availability

The data presented in this study are available on request from the corresponding author.

## Supplementary Materials

The following supporting information can be downloaded at: https://www.mdpi.com/article/10.3390/medicina60010122/s1. Table S1: Retrospective Data Chart of Study Participants
