# Supplementary material for: Acute Central Serous Chorioretinopathy Outbreak during the COVID-19 Pandemic: A Pilot Study
Source: Medicina (Kaunas). 2024 Jan 9;60(1):122. doi: 10.3390/medicina60010122 (PMC10818957; doi:10.3390/medicina60010122)
Supplement: Supplementary file 1 [file medicina-60-00122-s001.zip › medicina-2743789-supplementary.pdf]

Supplementary Table S1: Retrospective Data Chart of Study Participants

Case Number	Session Date	Year	COVID-19 Era	Period of Symptoms before Admission to Hospital (Days)	Visual Acuity (LogMAR)	CMT (microns)	Age	Gender (1=M)	Refractive Surgery	Myopia	Pseudophakia	Ischemic heart disease	Hypertension	SLE	BPH	Hyperlipidemia	ADHD	Malignancy in the Past	DM2	Glaucoma	Active smoking
1	22/02/2018	2018	0	60	0.10		49	1	0	0	0	0	0	0	0	0	0	0	0	0	0
2	07/10/2018	2018	0		0.18		59	1	0	0	0	1	0	1	0	0	0	0	0	0	0
3	26/03/2018	2018	0	1	0.08	310	70	1	0	0	0	0	0	0	0	0	0	0	0	0	0
4	15/05/2018	2018	0		0.30		48	0	0	0	0	0	0	0	0	0	0	0	0	0	0
5	22/03/2018	2018	0		0.18		48	1	0	0	0	0	0	0	0	0	0	0	0	0	0
6	24/01/2018	2018	0		0.48		60	1	0	0	0	1	0	0	0	0	0	0	0	0	0
7	09/12/2018	2018	0		0.18		56	0	0	0	0	0	0	0	0	0	0	0	0	0	0
8	08/05/2018	2018	0		0.18		33	0	0	0	0	0	0	0	0	0	0	0	0	0	0
9	13/06/2019	2019	0		0.30	371	49	1	0	0	0	0	0	0	0	0	0	0	0	0	0
10	16/09/2019	2019	0	14	0.18	577	34	1	0	0	1	0	0	0	0	0	1	0	0	0	1
11	26/12/2019	2019	0	14	0.18		49	1	1	1	0	0	0	0	0	0	0	0	0	0	0
12	07/11/2019	2019	0	30	0.18	516	41	1	0	0	0	0	0	0	0	0	0	0	1	0	0
13	30/05/2019	2019	0	14	0.18	601	41	1	0	0	0	0	0	0	0	0	0	1	0	0	0
14	17/11/2020	2020	1	21			26	1	1	1	0	0	0	0	0	0	0	0	0	0	0
15	24/06/2020	2020	1		0.40	687	53	0	0	0	0	1	1	0	0	0	0	0	0	0	1
16	30/08/2020	2020	1	3	0.10	515	33	1	0	0	0	0	0	0	0	0	0	0	0	0	0
17	13/05/2020	2020	1	14	1.00	455	37	1	0	0	0	0	0	0	0	0	0	0	0	0	0
18	15/09/2020	2020	1		1.00		50	1	0	0	0	0	0	0	0	0	0	0	0	0	0
19	08/07/2020	2020	1		0.10		52	1	0	0	0	0	0	0	0	0	0	0	0	0	0
20	20/05/2020	2020	1	30	0.10	423	55	1	0	0	0	0	0	0	1	0	0	0	0	0	0
21	30/08/2020	2020	1	1	0.30		37	1	0	0	0	0	1	0	0	0	0	0	1	0	0
22	12/10/2020	2020	1	30	0.70		40	1	0	1	0	0	0	0	0	0	0	0	0	0	1
23	09/06/2020	2020	1	14	0.30		57	1	0	0	0	0	0	0	0	0	0	0	0	0	0
24	15/11/2020	2020	1	180	0.30	389	36	1	0	0	0	0	0	0	0	0	0	0	0	0	0
25	15/11/2020	2020	1		0.10	483	36	1	0	0	0	0	0	0	0	0	0	1	0	0	0
26	02/07/2020	2020	1	3	0.10	460	38	1	0	1	0	0	0	0	0	0	0	0	0	0	1
27	10/05/2020	2020	1		0.18		64	1	0	0	0	0	0	0	0	1	0	0	0	0	0
28	16/12/2020	2020	1	2	1.00	414	32	1	1	1	0	0	0	0	0	0	0	0	0	0	0
29	11/01/2021	2021	1	75	0.30	370	42	0	0	0	0	0	0	0	0	0	0	0	0	0	1
30	11/01/2021	2021	1	30	1.00	581	40	1	1	1	0	0	0	0	0	0	0	0	0	0	0
31	07/04/2021	2021	0		0.4	370	42	0	0	0	0	0	0	0	0	0	0	0	0	0	0
32	05/05/2021	2021	0		0		40	1	0	0	0	0	0	0	0	0	0	0	0	0	0
33	19/06/2021	2021	0		0	590	34	1	0	0	0	0	0	0	0	0	0	0	0	0	0
34	11/07/2021	2021	0		0.48		41	1	0	0	0	0	0	0	0	0	0	0	0	1	0
35	16/09/2021	2021	0		0.1	678	45	1	0	0	0	0	0	0	0	0	0	0	0	0	0

CMT: Central Macular Thickness; SLE: Systemic Lupus Erythematosus; BPH: Benign Prostatic Hyperplasia ;ADHD: Attention Deficit Hyperactivity Disorder; DM2: Diabetes Mellitus type 2
